# TGF-β_1 _induced epithelial to mesenchymal transition (EMT) in human bronchial epithelial cells is enhanced by IL-1β but not abrogated by corticosteroids

**DOI:** 10.1186/1465-9921-10-100

**Published:** 2009-10-27

**Authors:** Astrid M Doerner, Bruce L Zuraw

**Affiliations:** 1 Veterans Medical Research Foundation, La Jolla, California, USA; 2 Department of Medicine, University of California, San Diego, La Jolla, California, USA

## Abstract

**Background:**

Chronic persistent asthma is characterized by ongoing airway inflammation and airway remodeling. The processes leading to airway remodeling are poorly understood, and there is increasing evidence that even aggressive anti-inflammatory therapy does not completely prevent this process. We sought to investigate whether TGFβ_1 _stimulates bronchial epithelial cells to undergo transition to a mesenchymal phenotype, and whether this transition can be abrogated by corticosteroid treatment or enhanced by the pro-inflammatory cytokine IL-1β.

**Methods:**

BEAS-2B and primary normal human bronchial epithelial cells were stimulated with TGFβ_1 _and expression of epithelial and mesenchymal markers assessed by quantitative real-time PCR, immunoblotting, immunofluorescence microscopy and zymography. In some cases the epithelial cells were also incubated with corticosteroids or IL-1β. Results were analyzed using non-parametric statistical tests.

**Results:**

Treatment of BEAS-2B or primary human bronchial epithelial cells with TGFβ_1 _significantly reduced the expression level of the epithelial adherence junction protein E-cadherin. TGFβ_1 _then markedly induced mesenchymal marker proteins such as collagen I, tenascin C, fibronectin and α-smooth muscle actin mRNA in a dose dependant manner. The process of mesenchymal transition was accompanied by a morphological change towards a more spindle shaped fibroblast cell type with a more motile and invasive phenotype. Corticosteroid pre-treatment did not significantly alter the TGFβ_1 _induced transition but IL-1β enhanced the transition.

**Conclusion:**

Our results indicate, that TGFβ_1 _can induce mesenchymal transition in the bronchial epithelial cell line and primary cells. Since asthma has been strongly associated with increased expression of TGFβ_1 _in the airway, epithelial to mesenchymal transition may contribute to the contractile and fibrotic remodeling process that accompanies chronic asthma.

## Background

Asthma is a chronic inflammatory disease of the airway, affecting approximately 10% of the general population [1]. Persistent asthma is characterized by structural changes termed airway remodeling. This ongoing remodeling and reconstruction of the asthmatic lung includes subepithelial fibrosis, myofibroblast hyperplasia, myocyte hyperplasia and/or hypertrophy, thickening of the lamina reticularis, and increased smooth muscle mass [2]. The more rapid decline in lung function over time in asthmatics is considered to be at least partly caused by this remodeling process. While the impact of corticosteroid treatment on airway remodeling is controversial, even aggressive anti-inflammatory therapy with corticosteroids does not appear to fully prevent remodeling and these long term effects [3]. It is important, therefore, to understand both the processes that contribute to remodeling in asthma as well as the impact of corticosteroids on these processes.

Myofibroblasts are considered a hallmark feature of the remodeling process in asthma. They are a morphological intermediate between fibroblasts and smooth muscle cells, and display increased synthetic activity [4]. Histologic examination of human asthmatic airways has revealed the presence of myofibroblasts in the proximity of both the smooth muscle layer and the lamina reticularis [5,6]. Due to their highly synthetic nature they are thought to contribute significantly to the thickening of the airway basement membrane. Myofibroblasts also express alpha-smooth muscle actin (αSMA), and therefore possess contractile properties similar to smooth muscle cells. Furthermore, myofibroblasts have been proposed to be capable of fully differentiating into smooth muscle cells thereby contributing to the increased smooth muscle mass observed in chronic asthma [7].

The origin of lung myofibroblasts has remained ill defined. Classically, myofibroblasts were thought to arise from the underlying fibroblast tissue [8,9]. Blood-circulating fibrocytes, which can home to the site of fibrotic tissue, have also been proposed as a source of lung myofibroblasts [10-12]. Recently, the hypothesis that myofibroblasts arise from epithelial cells through epithelial to mesenchymal transition (EMT) has been proposed [13-15]. EMT is a process in which epithelial cells may revert to synthetically active mesenchymal fibroblast-like cells, and is recognized as a crucial component of normal development [16]. In recent years it has been recognized, initially in epithelial cancer, that mature epithelial cells can undergo a second round of EMT, leading to a hyperactive and invasive, motile cell type.

In tubular epithelial cells in the kidney, EMT can be induced by TGFβ_1_, leading to increased collagen deposition and disruption of the epithelial integrity [17]. TGFβ_1 _is known to be expressed by a variety of inflammatory and structural lung cells in asthma, and is also recognized to be involved in lung fibrosis. Recent publications in the field of idiopathic pulmonary fibrosis (IPF) also point to the alveolar epithelium as a major contributor to fibrosis by undergoing EMT [13,15,18]. Studies employing the cancer derived human alveolar epithelial cell line, A549, have confirmed the ability of alveolar epithelial cells to undergo EMT *in vitro *[14]. Less is known, however, regarding the ability of human bronchial epithelial cells to undergo EMT. In a recent study of obliterative bronchiolitis (OB) in chronic rejection of lung allografts, Ward et al. [19] showed compelling evidence for EMT occurring in bronchial airway epithelial cells in vivo, suggesting a link between injury and remodeling. While there has been no clear evidence that EMT occurs in patients with asthma, Hackett et al. demonstrated that TGFβ_1 _induces EMT in both normal and asthmatic primary bronchial epithelial cells *in vitro *[20].

Although, the regulation of TGFβ_1 _in asthma remains incompletely understood, many investigators have reported increased TGFβ_1 _levels in asthma. Compared to normal subjects, asthmatic subjects were found to have elevated TGFβ_1 _levels in bronchoalveolar lavage (BAL) fluid and bronchial biopsies [21,22]. The increase in TGFβ_1 _was shown to persist despite oral corticosteroid treatment [22,23] and to correlate with basement membrane thickness and fibroblast number [24].

We hypothesized that bronchial epithelial cells may also undergo EMT during chronic asthmatic inflammation, thereby providing an additional source for myofibroblasts, and contributing to the remodeling process observed in the asthmatic lung. Here we report evidence, that TGFβ_1 _induces EMT in the bronchial epithelial cell line BEAS-2B as well as in primary normal human bronchial epithelial cells (NHBE). We further demonstrate that IL-1β may assist in EMT by initiating crucial changes in protein expression pattern. Pre-treatment with corticosteroids inhibited some of the EMT changes but had no impact on the majority of changes. Our findings suggest that bronchial epithelial cells do undergo TGFβ_1_-induced EMT and synthesize matrix proteins, and that corticosteroid treatment does not completely prevent this process. Bronchial epithelial cell EMT may thus be a significant contributor to the contractile and fibrotic remodeling process that accompanies chronic asthma.

## Methods

### Cell culture

Primary NHBE (Lonza, Wakersville, MD) and transformed human bronchial epithelial cell line BEAS-2B (CRL-9609; American Type Culture Collection, Manassas, VA) were grown as monolayers in 100% humidity and 5% CO_2 _at 37°C in serum-free defined growth media (BEGM, Lonza) or keratinocyte media (Invitrogen, Carlsbad, CA). NHBEs were used on passage 2 or 3. NHBE and BEAS-2B cells were seeded a day prior to starting the treatment at ~30-40% confluence in 6 well or 12 well plates, then stimulated with recombinant human TGFβ_1 _(R&D Systems, Minneapolis, MN) and/or IL-1β (R&D Systems, Minneapolis, MN) in complete medium at the indicated concentrations or complete medium alone. Dexamethasone (10^-7^M) or budesonide (10^-8^M) (Sigma-Aldrich, St. Louis, MO) were added to the medium 16 h before stimulation with TGFβ_1 _(1 ng/ml). Medium with or without TGFβ_1 _was changed every 2 days. The experiments were designed so that the cells for all time points reached confluence one day prior to harvesting. Cells were therefore seeded and harvested at the same time, but the cytokines or corticosteroids were added at the appropriate times for the individual time points. Cells were lysed in RLT buffer (Qiagen, Valencia, CA) or RNA Stat 60 (Tel-Test, Friendswood, TX) reagent respectively for RNA isolation or in protein lysis buffer.

### RNA isolation, reverse transcription and quantitative real-time PCR

Total RNA was extracted as previously described [25]. The ABI 7300 real-time PCR machine (Applied Biosystems, Foster City, CA) was used for real-time quantitative PCR. The specific primers and dual labeled probes (Biosearch technologies, Novato, CA) used in the real-time PCR are listed in Table 1. The starting amount of cDNA in the samples was calculated using the ABI software package (Applied Biosystems, FosterCity, CA).

**Table 1:** Real-time PCR primer and probe sequences

Target	Sense primer (5' ⇒ 3')	Antisense primer (5' ⇒ 3')	Probe (5'FAM ⇒ 3'BHQ)
E-cadherin	CCACCAAAGTCACGCTGAATAC	GGAGTTGGGAAATGTGAGCAA	CCATCAGGCCTCCGTTTCTGG

α-SMA	CTGGCATCGTGCTGGACTCT	GATCTCGGCCAGCCAGATC	ATGCCTTGCCCCATGCCATCA

Tenascin C	CAGAAGCCGAACCGGAAGTT	TTCATCAGCTGTCCAGGACAGA	TGCCACCCCAGACGGTTTCC

Fibronectin-EDA	GAGCTATTCCCTGCACCTGATG	CGTGCAAGGCAACCACACT	TGCAAGGCCTCAGACCGGGTTC

Collagen I	CCTCAAGGGCTCCAAC	GGTTTTGTATTCAATCACTGTCTTGC	ATGGCTGCACGAGTCACACCGGA

Vimentin	GGAAGAGAACTTTGCCGTTGAA	GTGACGAGCCATTTCCTCCTT	CCAAGACACTATTGGCCGCCTG

β-actin	TGCGTGACATTAAGGAGAAG	GTCAGGCAGCTCGTAGCTCT	CACGGCTGCTTCCAGCTCCTC

2-microglobulin	AGCGTCTCCAAAGATTCAG	AGACACATAGCAATTCAGGA	ACTCACGTCATCCAGCAGAGAATGG

### Protein isolation and immunoblotting

Protein isolation and immunoblotting were performed as previously described [26] using 20 μg of total protein and nitrocellulose membrane. Specific antibodies were used at a dilution of 1:500 for the detection of α SMA (mouse anti-human clone 1A4, Sigma) or 1:1000 for E-cadherin (rabbit anti-human, H-108, Santa Cruz Biotechnology Inc., Santa Cruz, CA) or 1:500 for fibronectin (mouse anti-human ascites fluid, clone IST-4, Sigma), followed by horseradish peroxidase (HRPO)-conjugated goat anti-rabbit or goat anti-mouse antibodies respectively. Immunoblotting for β-actin (specific IgM antibody, a gift from Dr Ed Chan, Dept. of Molecular and Experimental Medicine, TSRI, La Jolla, USA) was used as loading control.

### Wound healing and invasion assay

BEAS-2B cells were seeded in 6-well plates and 16 h later stimulated with 5 ng/ml TGFβ_1 _or complete medium alone for 3 days. Wells were marked with a straight black line on the bottom for orientation later. Cells were ~90% confluent at the time of scratch wounding. Three scratch wounds were applied in each well with a 200 ul pipette tip and non-adherent cells washed off with medium. Fresh medium with or without TGFβ_1 _was added to the wells and cells were incubated for up to 48 h. Phase contrast light microscope pictures were taken on an EVOS inverted microscope from AMG immediately after scratch wounding (0 h), at 24 h and 48 h. Pictures were aligned using the orientation line to ensure that the identical spots were followed over time. Experiments were conducted independently 3 times each in triplicate.

BEAS-2B cells were seeded in T25 flasks and stimulated for 4 days with or without TGFβ_1 _in complete medium. Cells were harvested and seeded at 50.000 cells per well on Matrigel™ coated inserts (24 well BioCoat™ Matrigel™ invasion chamber, 8 um pores, BD Bioscience) in complete medium without adding TGFβ_1_. After 24 h incubation, cells were swiped off the top of the inserts and cells that penetrated the filters were stained with Protocol Hema 3 (Fisher Diagnostics). The number of invasive cells was determined by counting all cells attached to the bottom of the inserts under a light microscope at 10× magnification. Experiments were conducted independently 3 times each in triplicate.

### Gelatin zymography for matrix metalloproteinases expression

NHBE and BEAS-2B cells were stimulated with TGFβ_1 _(1 or 5 ng/ml) in complete media for up to 4 days without changing the media. One ml of fresh media was added after 2 days of stimulation. 20 μl of conditioned media were subject to zymography as described elsewhere [17] using buffers from Bio-Rad. Protein bands were visualized according to the manufactures manual. Protein bands appear white in blue background.

### Immunofluorescence staining for E-cadherin

BEAS-2B cells were grown on rat tail-collagen I coated glass coverslips (22 mm, BD Bioscience, Bedford, MA) and stimulated with TGFβ_1 _(5 ng/ml) for 4 days as described above. Coverslips were stained with monoclonal mouse anti-E-cadherin antibody (R&D Systems, Minneapolis, MN) in a dilution of 1:200, followed by the secondary antibody (goat anti-mouse conjugated with Alexa488, Jackson ImmunoResearch Laboratories Inc., West Grove, PA) in a dilution of 1:300. As a negative control the primary antibody was omitted. Nuclei were stained with 4',6-diamidino-2-phenylindole (DAPI) (Sigma-Aldrich, St. Louis, MO) and coverslips mounted with Fluoromount-G (Southern Biotech, Birmingham, AL). Images were captured with an Olympus Fluoview 1000 laser scanning confocal microscope (Olympus BX61 microscope equipped with a x20/0.7 dry objective lens and Fluoview acquisition software; Olympus, Tokyo, Japan) and the two channels merged in the Olympus Fluoview software.

### Statistical Analysis

Data were analyzed by the non-parametric Kruskal-Wallis one-way analysis of variance or non-parametric Mann-Whitney U tests.

## Results

### TGFβ_1 _induces morphological changes in bronchial epithelial cells

**Figure 1:**
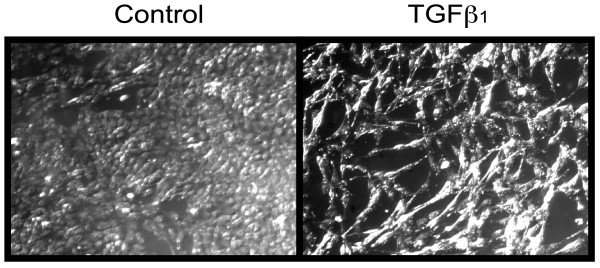
**Morphological changes induced by TGFβ_1_**. BEAS-2B cells were grown to 40% confluency in tissue culture plates and stimulated with TGFβ_1 _(5 ng/ml) or complete medium alone (*control*) for 3 days. Pictures were taken with bright field illumination using a Leica DM IRB inverted microscope equipped with a Hamamatsu digital camera and processed with OpenLab 3.1.7 image acquisition software.

Stimulation of the bronchial epithelial cells line BEAS-2B with TGFβ_1 _induced a change of morphology consistent with EMT (Figure 1). Cells stimulated with TGFβ_1 _developed a spindle fibroblast-like morphology with reduced cell-cell contact, while cells in media alone maintained the typical epithelial cobblestone pattern.

### TGFβ_1 _induces gene expression characteristic of EMT

**Figure 2:**
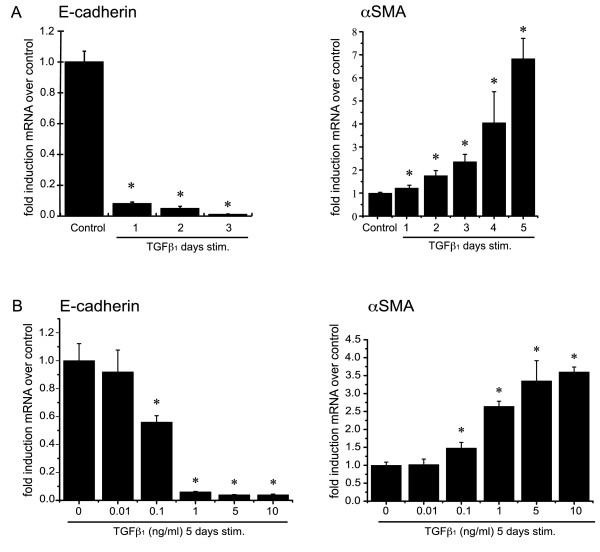
**Expression changes of E-cadherin and αSMA in BEAS-2B cells upon TGFβ_1 _treatment**. BEAS-2B cells were stimulated with TGFβ_1 _or complete medium alone (*control*) in triplicate for the indicated time and doses. Total RNA was isolated and assessed in triplicate for the expression of E-cadherin, α SMA and β-actin by means of quantitative real-time PCR. Expression levels were normalized to the housekeeping gene β-actin and calculated as mean level of induction in comparison to control untreated cells. **A**: Time course: Beas-2B cells were stimulated with 5 ng/ml TGFβ_1 _from one to 5 days. (*p < 0.01 by Kruskal-Wallis one-way ANOVA). **B**: Dose response: Beas-2B cells were treated with TGFβ_1 _from 0.001 ng to 10 ng/ml for 5 days (*p < 0.05 compared to control by Mann-Whitney *U *test).

EMT is defined by changes in gene expression in which epithelial markers such as E-cadherin decrease while mesenchymal markers such as αSMA (a marker characteristic for myofibroblasts) increase. BEAS-2B cells were stimulated with TGFβ_1 _5 ng/ml and E-cadherin and αSMA expression quantified by quantitative real time PCR. TGFβ_1 _significantly reduced E-cadherin mRNA levels while simultaneously increasing expression of αSMA (Figure 2A).

We determined the minimal concentration of TGFβ_1 _sufficient to induce EMT in BEAS-2B cells. Expression of E-cadherin and αSMA mRNA were determined after treating the cells with TGFβ_1 _in a dose range from 0.01 ng/ml to 10 ng/ml. Concentrations as low as 0.1 ng/ml TGFβ_1 _were sufficient to induce the phenotypic markers of EMT with the maximal response at 1 ng/ml for both genes (Figure 2B).

**Figure 3:**
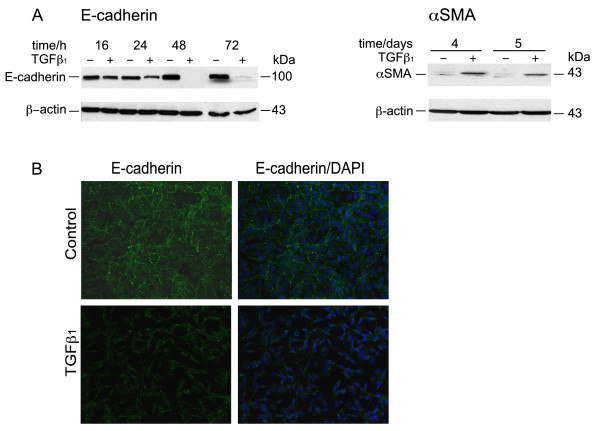
**Changes of E-cadherin and αSMA protein levels in BEAS-2B cells upon TGFβ_1 _treatment**. **A**: Cell lysates from BEAS-2B cells, stimulated for the indicated time with TGFβ_1 _(5 ng/ml) or complete medium, were immunoblotted for E-cadherin or αSMA as described in Methods. Blots were stripped and rehybridized with an antibody to β-actin. **B**: Immunofluorescent staining for E-cadherin in BEAS-2B cells stimulated with TGFβ_1 _(5 ng/ml) for 4 days or complete medium alone. The left panel shows only the E-cadherin fluorescence (pseudocolor green) and the right panel the overlay with the DAPI fluorescence (pseudocolor blue). Images were captured at a magnification of 20×. Results are representative of 3 separate experiments.

To confirm these mRNA changes, we assessed the effects of TGFβ_1 _on E-cadherin and αSMA protein levels in BEAS-2B cells (Figure 3A). Immunoblotting of cell lysates demonstrated that E-cadherin protein levels fell within 24 h of incubation with TGFβ_1_. While not normally expressed by BEAS-2B cells, αSMA protein became detectable after 4 days of TGFβ_1 _treatment. The decrease in cell-cell contact induced by TGFβ_1 _was also confirmed by immunofluorescence staining for E-cadherin that demonstrated a loss of the grid-like localization of E-cadherin at the cell-cell contact surface following TGFβ_1 _treatment (Figure 3B).

### TGFβ_1 _stimulates the expression of basement membrane proteins relevant for fibrogenesis in epithelial cells

**Figure 4:**
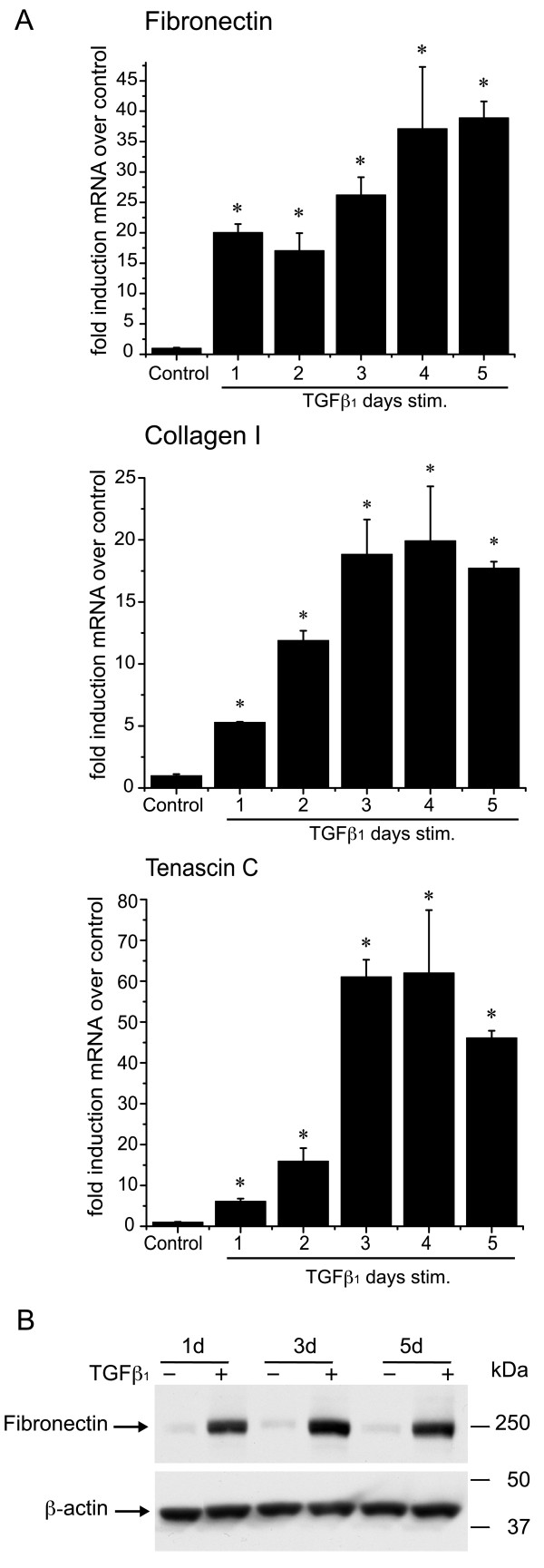
**TGFβ_1 _increases the expression of connective tissue proteins in BEAS-2B cells**. **A: **BEAS-2B cells were stimulated with TGFβ_1 _(5 ng/ml) or complete medium alone (*control*) and the levels of fibronectin-EDA, collagen I and tenascin C mRNA were analyzed by quantitative real-time PCR as described in Fig 2A. (*p < 0.05 by Kruskal-Wallis one-way ANOVA) **B: **Cell lysates from BEAS-2B cells, stimulated for the indicated time with TGFβ_1 _(5 ng/ml) or complete medium, were immunoblotted for fibronectin as described in Methods. Blots were stripped and rehybridized with an antibody to β-actin.

Asthma is accompanied by the thickening of the basement membrane due to the excessive production of matrix proteins typically synthesized by fibroblasts and myofibroblasts, including collagen I and III as well as fibronectin-EDA and tenascin C. Our results shown above suggested that bronchial epithelial cells can transition into a mesenchymal-like phenotype upon TGFβ_1_treatment and might therefore contribute to deposition of excessive matrix proteins. Within 24 hrs following stimulation with TGFβ_1_, BEAS-2B cells demonstrated significant increases in the mRNA expression of collagen I, fibronectin-EDA and tenascin C (Figure 4A). Synthesis of fibronectin was also measured at the protein level, and was found to be significantly increased by treatment with TGFβ_1 _(Figure 4B). Expression of collagen III mRNA, unlike collagen I, was not changed by TGFβ_1 _(data not shown).

### TGFβ_1 _stimulation increases migration, invasion and release of MMP-2 and MMP-9 proteins

**Figure 5:**
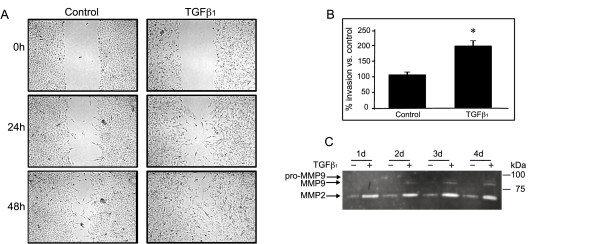
**TGFβ_1 _increases migration and invasion of BEAS-2B cells**. **A**: BEAS-2B cells pre-stimulated with TGFβ_1 _(5 ng/ml) or complete medium alone for 3 days, followed scratch wounding with a 200 ul pipette tip at ~90% confluence. Pictures of the same area were taken under bright field illumination immediately after wounding (0 h) as well as 24 h and 48 h later. **B: **BEAS-2B cells were pre-stimulated with TGFβ_1 _(5 ng/ml) or complete medium alone for 4 days, seeded on Matrigel coated inserts in complete medium without the addition of TGFβ_1 _and incubated for 24 h. Epithelial cells, which had migrated through the inserts were counted under light microscope at 10× magnification. The number of invading cells after TGFβ_1 _treatment were normalized to the number of invading control untreated cells, which were set as 100%. Data are averaged from three independent experiment each performed triplicate. (*p < 0.0001 compared to control by unpaired Wilcoxon-Mann-Whitney Rank Sum Test). **C: **Conditioned media of BEAS-2B cells stimulated with TGFβ_1 _(5 ng/ml) or complete medium alone were subject to gelatin-zymography. Experiments were conducted three times with similar results.

EMT has been linked to increased migration and invasiveness in the context of cancer [27,28] as well as in complications associated with lung transplants [19,29]. We therefore first assessed the capability for migration of BEAS-2B cells with or without TGFβ_1 _pre-treatment in a scratch-wound healing assay. Cells pre-treated with TGFβ_1 _for 3 days showed much higher motility and achieved almost complete wound closure within 48 h in contrast to untreated cells (Figure 5A). Next, we assessed the effect of TGFβ_1 _on cell invasion. In an invasion assay utilizing Matrigel™ coated cell inserts we observed up to 100% increased invasion by cells pre-treated for 4 days with TGFβ_1 _in comparison to untreated cells (Figure 5B).

Since increased expression of matrix-metalloproteinases has been observed in EMT and connected to enhanced cell migration and invasiveness, we then assessed the expression of matrix-metalloproteinases (MMP), specifically MMP2 and MMP9 by gelatin zymography. Supernatants from unstimulated BEAS-2B cells showed a low basal level of MMP2 protein, which was significantly up-regulated within 24 h of TGFβ_1 _treatment (Figure 5C). MMP-9 protein levels were undetectable at baseline levels, but increased by 48 h to 96 h of treatment. MMP-9 was detected as a double band corresponding to the zymogen, pro-MMP-9 protein, at 92 kDa and the cleaved mature form at 86 kDa.

### TGFβ_1 _stimulation increases expression of EMT markers in primary normal human bronchial epithelial cells

**Figure 6:**
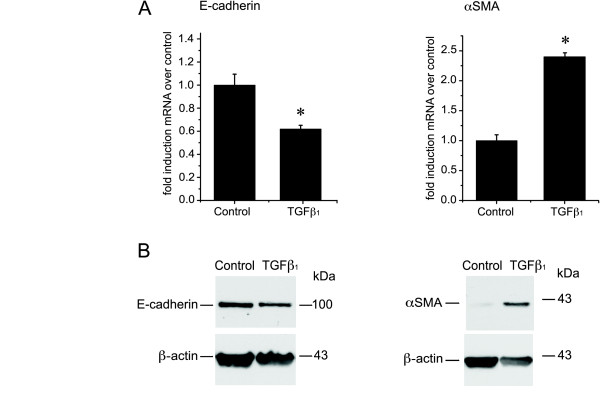
**TGFβ_1 _increases the mRNA of EMT-marker proteins in primary human bronchial epithelial cells (NHBE)**. **A**: NHBE cells were stimulated with TGFβ_1 _(2 ng/ml) or complete medium alone (*control*) for 3 days in triplicate. The levels of αSMA and E-cadherin mRNA were analyzed by quantitative real-time PCR. Expression levels were normalized to the housekeeping gene 2-microglobulin and calculated as fold induction in comparison to control. Results are representative of experiments performed with 2 different donors. (*p < 0.05 compared to control by Mann-Whitney *U *test) **B**: Cell lysates from NHBE cells, stimulated for the indicated time with TGFβ_1 _(2 ng/ml) or complete medium, were immunoblotted with for E-cadherin or αSMA. Blots were reprobed for β-actin as loading control.

**Figure 7:**
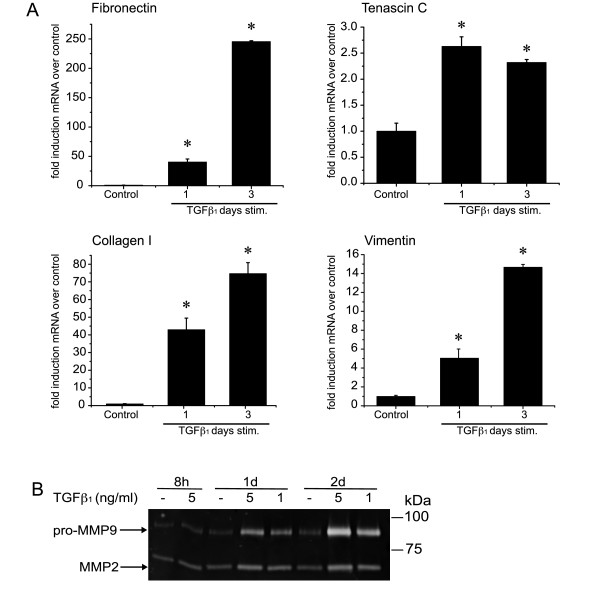
**TGFβ_1 _induces the expression of EMT-marker proteins and matrix-metalloproteinases in NHBE**. **A**: NHBE cells were stimulated with TGFβ_1 _(2 ng/ml) or complete medium alone (*control*) for one to 3 days in triplicate. The levels of fibronectin-EDA, tenascin C, collagen I and vimentin mRNA were analyzed by quantitative real-time PCR as described in figure 5A. Results are representative of experiments performed with 2 different donors. (*p < 0.05 compared to control by Mann-Whitney *U *test) **B**: Conditioned supernatant of NHBE cells stimulated with TGFβ_1 _(5 ng/ml or 1 ng/ml) or complete medium alone were subject to Zymography. Results are representative of 2 separate experiments performed with 2 different donors.

Since BEAS-2B cells are a transformed human bronchial epithelial cell line, we then assessed whether primary NHBE cells also undergo EMT in response to TGFβ_1 _(Figure 6 + 7). Preliminary dose response experiments revealed that the NHBE cells were more sensitive to TGFβ_1_-induced apoptosis (data not shown). The TGFβ_1 _dose used in these experiments was therefore reduced from 5 ng/ml to 2 ng/ml. Like the pattern observed in BEAS-2B cells, TGFβ_1 _induced a fall in E-cadherin and an increase in αSMA mRNA (Figure 6A) in the NHBE cells as well as the corresponding changes in the protein levels (Figure 6B). TGFβ_1 _stimulation of NHBE cells also induced increased mRNA levels for fibronectin, tenascin C and collagen I (Figure 7A), as well as increased MMP-2 and MMP-9 activities in the culture supernatant (Figure 7B). TGFβ_1 _induced an increase of vimentin mRNA in NHBEs (Figure 7A), an effect that was not observed in BEAS-2B cells. This difference in the expression profile might be due to variances between primary cells and transformed cell lines. Experiments were repeated using NHBE derived from two different donors. Both donors showed similar results with only minor variations in the time course and magnitude of change in mRNA expression.

### IL-1β reduces the expression of E-cadherin and enhances the effects of TGFβ_1 _on tenascin C expression

**Figure 8:**
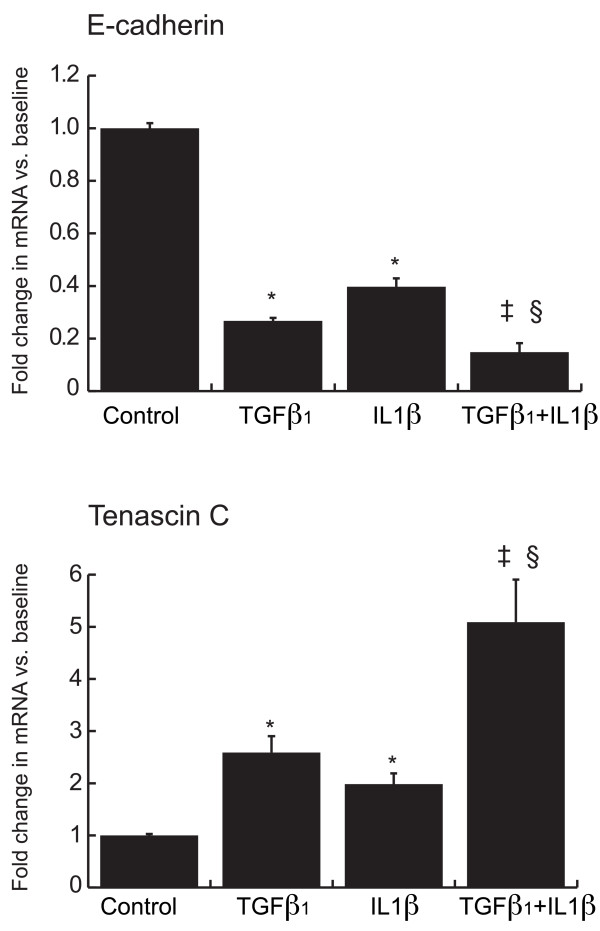
**IL-1β reduce the expression of E-cadherin and enhances the effects of TGFβ on Tenascin C expression**. BEAS-2B cells were stimulated with TGFβ_1 _(0.1 ng/ml) or IL-1β (1 ng/ml) or both for 3 days in triplicate. Total RNA was isolated and assessed in triplicate for the expression of E-cadherin and Tenascin C by means of quantitative real-time PCR. Expression levels were normalized to the housekeeping gene β-actin and calculated as mean level of induction in comparison to control untreated cells. Results show mean ± standard error of 3 separate experiments, each in triplicate (* p < 0.0001 compared to control; ‡ p < 0.02 compared to TGFβ_1 _treated; §p < 0.0005 compared to IL-β treated; all by Mann-Whitney *U *test).

We then examined whether the proinflammatory cytokine, IL-1β, could also induce EMT in bronchial epithelial cells. BEAS-2B cells were stimulated for 3 days with IL-1β, TGFβ_1_, the combination of IL-1β plus TGFβ_1_, or media alone then assessed for evidence of EMT (Figure 8). Similar to TGFβ_1_, IL-1β induced a significant decrease in E-cadherin expression and a significant increase in tenascin C expression. Unlike TGFβ_1 _however, IL-1β had no effect on the expression of αSMA or any of the other basement membrane proteins assessed (data not shown). When added together with TGFβ_1_, IL-1β had a significant additive impact on the decrease in E-cadherin and the increase in tenascin C expression compared to adding the cytokines individually. IL-1β had no additional impact on TGFβ_1_-induced changes in αSMA or other basement membrane proteins.

### Corticosteroid pretreatment does not abrogate TGFβ_1 _induced EMT

**Figure 9:**
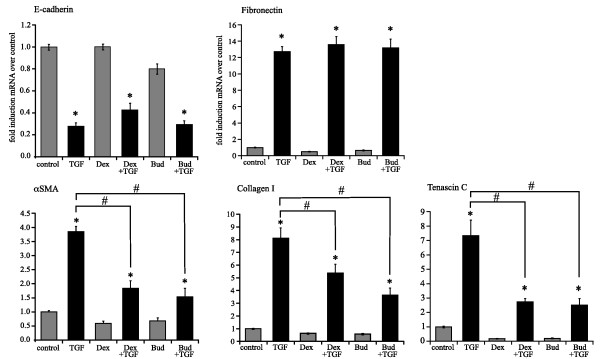
**Corticosteroids do not completely prevent TGFβ_1 _induced EMT**. BEAS-2B cells were pretreated with dexamethasone (dex, 10^-7 ^M) or budesonide (bud, 10^-8 ^M) for 16 hours followed by subsequent stimulation with TGFβ_1 _(1 ng/ml) for one or 3 days. mRNA expression levels were assessed by quantitative realtime PCR for E-cadherin (1 day) and αSMA, collagen I, fibronectin-EDA and tenascin C after 3 days. Results are representative of 3 separate experiments, each in triplicate. (*p < 0.05 compared to non-TGFβ_1 _stimulated controls by Mann-Whitney *U *test).

BEAS-2B cells were pretreated with dexamethasone or budesonide for 16 h and subsequently stimulated with TGFβ_1 _for 3 days. The dose of TGFβ_1 _used in these experiments was reduced to 1 ng/ml in order to enhance our ability to detect any corticosteroid effect. Analysis of the EMT marker genes revealed, that corticosteroid treatment had a variable but incomplete effect on TGFβ_1_-induced EMT (Figure 9). Corticosteroids did not substantially alter TGFβ_1_-mediated downregulation of E-cadherin mRNA or upregulation of collagen I, fibronectin, or tenascin mRNA. Budesonide and dexamethasone did, however, partially abrogate TGFβ_1 _induced αSMA mRNA upregulation. To confirm biologic activity of the corticosteroids, we determined the protein levels of GILZ [25] at 30 h of treatment. Dexamethasone and budesonide both potently upregulated GILZ as we reported earlier (data not shown).

## Discussion

Chronic asthma may be accompanied by an enhanced rate of decline in lung function irrespective of anti-inflammatory treatment. These clinical observations have been linked to structural changes in the asthmatic lung termed airway remodeling [30-32]. The pathogenesis of airway remodeling has been previously attributed to reactivation of the epithelial-mesenchymal trophic unit in which increased levels of TGFβ_1 _contribute to a state where hypo-proliferative but activated epithelial cells induce activation of fibroblasts to myofibroblasts [33-35]. Although TGFβ_1 _functions as a master switch in tissue repair and wound healing, there is substantial evidence that disordered expression of TGFβ_1 _may lead to fibrosis [15,36,37]. Clinical studies indeed confirm evidence for epithelial shedding and damage in the asthmatic airway, along with elevated levels of TGFβ_1 _in asthmatic bronchoalveolar lavage fluid and airway tissue [21,24]. While not all studies have found elevated TGFβ_1 _levels in the airways of asthmatic subjects [38,39], the bulk of evidence suggests that chronic asthmatic inflammation is accompanied by increased activity of TGFβ_1 _in the airways [21-23].

By virtue of their synthetic and contractile phenotype, myofibroblasts are considered to be a key cell type responsible for the excessive extracellular membrane protein deposition and increase in smooth muscle mass associated with remodeled airways [36,40]. The origin of the lung myofibroblast, however, is still unclear. An unknown percentage of lung myofibroblasts derive from activation of tissue fibroblasts or homing of blood-borne fibrocytes [11,41]. In addition, there is emerging evidence in kidney fibrosis and IPF that TGFβ_1_-driven EMT of tubular interstitial epithelial cells and alveolar epithelial cells may represent a significant source of tissue myofibroblasts [14,15,42,43]. TGFβ_1 _has previously been shown to induce EMT in the alveolar-type cancer cell line, A549 [14]. In addition, *in vivo *studies have suggested that EMT may occur in IPF as well as in alveolar and bronchial epithelial cells during bleomycin-induced pulmonary fibrosis [15,44,45].

Despite the accumulating evidence that EMT contributes to fibrotic remodeling in several organs including the lungs, there is little evidence that EMT occurs in bronchial epithelial cells and no evidence that it plays a role in the airway remodeling that accompanies chronic asthma. We hypothesized that exposure of normal bronchial epithelial cells to chronic TGFβ_1 _stimulation would cause them to undergo EMT, potentially representing another source of myofibroblasts involved in airway remodeling in asthma. Here we report that BEAS-2B as well as primary normal human bronchial epithelial cells show evidence of EMT upon prolonged *in vitro *stimulation with TGFβ_1_.

TGFβ_1_-induced downregulation of the epithelial cell specific adherence junction protein E-cadherin at both the mRNA and protein levels was the earliest effect we observed, reaching near-maximal effect within 24 hours of stimulation in BEAS-2B cells. The loss of cell-cell contact has been shown to be a crucial first event in the remodeling process in the kidney [17,46]. Masszi [47] et al. further reported that the disruption of cell-cell contact is a critical regulator for TGFβ_1 _induced EMT in kidney cells. They suggest a two-hit mechanism in which both TGFβ_1 _stimulation as well as initial epithelial injury are required for the induction of EMT. This correlates with the observation that in the asthmatic airway the integrity of the epithelial layer is disrupted, which might therefore facilitate the fibrogenic action of TGFβ_1_. Further it has been demonstrated that β-catenin, released from the cytosolic portion of E-cadherin, can function as a transcription factor in concert with the lymphoid enhancing factor 1 (LEF1) and induces EMT in epithelial cell lines [47-49].

Myofibroblasts release a variety of ECM proteins contributing to the thickening of the lamina reticularis, a key feature in the remodeling process of the lung. We found that TGFβ_1 _stimulates increased expression of extracellular matrix proteins (fibronectin, collagen I and tenascin C) in BEAS-2B cells. Stable expression of a myofibroblast phenotype in renal epithelial cells has been shown to depend on both TGFβ_1 _and adherence signals [13,46,50,51]. In this regard, TGFβ_1 _induced expression of fibronectin and integrins appeared to be necessary for the subsequent induction of the expression of αSMA in renal cells [51,52].

Because EMT results in an increase in cell migration and invasiveness, we assessed the migratory and invasive capacity of TGFβ_1 _exposed BEAS-2B cells. We observed both increased migration and enhanced invasiveness in BEAS-2B cells subjected to chronic exposure to TGFβ_1_, similar to the results reported by Borthwick et al [29] of epithelial cells undergoing EMT in the context of obliterative bronchiolitis (OB) following lung transplantation. The acquisition of a more motile phenotype of bronchial epithelial cells undergoing EMT might facilitate invasion of the sub-epithelial layer with enhanced contribution to the deposition of excess matrix proteins. Accompanying the increased migratory and invasive phenotype, we also observed elevated production and secretion of MMP-9 and MMP-2. MMP-2 and MMP-9 not only promote a motile cell phenotype through matrix degradation but can also activate latent TGFβ_1_. Induction of MMP-2 expression has been reported to be an important step in kidney fibrosis by disrupting the basement membrane thereby facilitating the migration of epithelial derived myofibroblasts into the interstitium [17]. Further, asthmatic patients have increased immunoreactivity for MMP-9 in their airway epithelium and submucosa [53,54]. Overexpression of MMP-proteins could further contribute to airway remodeling in asthma by feeding into the cycle of excess production and turn-over of matrix proteins. A correlation between fibrosis in asthma and MMP-9 expression has recently been demonstrated in a mouse model of chronic asthma [55]. MMP-9 knockout mice showed a modest reduction in fibrosis, although no effect on mucus production or smooth muscle thickness was observed, suggesting a restricted role of MMP-9 in airway remodeling.

Alpha smooth muscle actin is characteristically expressed in myofibroblasts, enabling contractibility and an overall more invasive motile cell type. We detected upregulation of αSMA on the mRNA as well as protein level in BEAS-2B by days 3 to 4. In a recent clinical study Larsen et al [56] showed evidence for activated mobile fibroblasts in the BAL fluid of mild asthmatics, which upon stimulation with TGFβ_1 _produced more ECM proteins. These *in vivo *data are consistent with our hypothesis that epithelial cells undergo transition into myofibroblasts in the context of asthma.

Our results using BEAS-2B cells show that TGFβ_1 _clearly induces EMT in the transformed bronchial epithelial cell line. Importantly, we also observed an almost identical pattern of EMT following stimulation with TGFβ_1 _in primary normal human bronchial epithelial cells (NHBE). These results establish that TGFβ-induced EMT is not limited to alveolar epithelial cells but can also be induced in normal human bronchial epithelial cells *in vitro*. It is important to note, however, that our experiments utilized normal rather than asthmatic cells. Wound healing is part of the normal response of the epithelium to injury. In asthma the chronic cycle of injury and repair is thought to lead to the deregulation of factors involved, resulting in airway remodeling [57]. Our observations further highlight the possible functional consequences of EMT in both physiologic wound healing as well as pathophysiologic remodeling in the airway. We also studied cells cultured under submerged conditions rather than at the air-liquid interface. A recent report by Hackett et al. [20] did study TGFβ_1 _induced EMT in both primary airway epithelial cells from normal and asthmatic donors as well as grown under submerged versus air-liquid interface conditions. They observed no differences between normal and asthmatic cells under submerged conditions. Under air-liquid interface conditions, the only significant difference they observed was that EMT was restricted to the basal cells in normal cultures but was less restricted in asthmatic cultures.

The inflammatory cytokine IL-1β is elevated in BAL fluid of symptomatic asthmatics [58], and there is evidence for a cross-talk between the TGFβ_1 _and IL-1β signaling pathways [59]. Furthermore, overexpression of IL-1β caused emphysema and fibrosis in the airway walls in a murine model of COPD [60] and IL-1β has been shown to induce endothelial to mesenchymal transformation in skin [61]. Therefore, we assessed the impact of IL-1β on TGFβ_1_-induced EMT in BEAS-2B cells. By itself, IL-1β induced a statistically significant decrease in E-cadherin expression and a statistically significant increase in tenascin C expression. When added together with TGFβ_1_, IL-1β had a significant additive effect on the changes in expression of these genes. Considering the critical role decreased E-cadherin plays in the initiation of EMT, the limited effect of IL-1β may prove to be biologically significant. Our results are compatible to the report by Kim et al. [62] showing synergistic effects of TGFβ_1 _and IL-1β on the expression of mesenchymal markers in the A549 cancer cell line, without evidence of induction of EMT by IL-1β alone.

Bronchial asthma is a chronic inflammatory disorder in which corticosteroids have become the first line of therapy. Whereas multiple studies support the benefit of corticosteroid treatment in respect to asthma symptoms and disease exacerbations [53,63], there is considerable uncertainty concerning whether corticosteroids significantly slow airway remodeling. Several clinical studies as well as studies using murine models of allergic airway inflammation have suggested that corticosteroids reduce subepithelial fibrosis [19,63-66]. Other clinical studies show evidence for persistently elevated levels of TGFβ_1 _and peribronchial fibrosis in the airway of asthmatic patients despite the reduction of inflammatory cells following treatment with corticosteroids [22,67].

We were therefore interested to test the impact of corticosteroids in our model of EMT. Preincubation of BEAS-2B cells with dexamethasone or budesonide followed by TGFβ_1 _stimulation in a moderate concentration did not prevent the morphological changes or influence the reduction in E-cadherin expression. The effect on the induction of ECM proteins was variable as we observed no reduction of the TGFβ_1 _induced expression of fibronectin-EDA, and a slight reduction in the expression of collagen I and tenascin C. Budesonide, but not dexamethasone, inhibited TGFβ_1 _induced αSMA expression. We did not confirm the lack of efficacy of corticosteroids in abrogating EMT using primary airway epithelial cells, and this will be important to do in future studies.

These data suggest that corticosteroid have only a modest impact on TGFβ_1_-induced EMT. This finding is consistent with reports that while corticosteroid have proven to be very beneficial in treating asthmatic inflammation, their efficacy in preventing or reversing the remodeling process may be limited. New therapy strategies may need to be developed to target airway remodeling in asthma. TGFβ_1 _has been proposed as a target using anti-sense oligonucleotide, pan specific neutralizing antibodies as well as kinase inhibitors targeting TGFβ_1 _receptors. Anti-TGFβ_1 _and TGFβ_2 _antibodies have been shown to be effective in animal models of renal and ocular fibrosis and are currently in phase I/II trials in humans (reviewed in [68]).

## Conclusion

In summary, we show evidence that human bronchial epithelial cells undergo EMT upon chronic TGFβ_1 _stimulation, that IL-1β enhances TGFβ_1_-induced EMT, and that corticosteroids do not substantially abrogate these effects. Additional studies are clearly needed to both confirm these results in primary asthmatic airway epithelial cells and address whether EMT occurs *in vivo *during asthmatic inflammation. Based on our results we suggest that bronchial epithelial cells might be one source for myofibroblasts in vivo in the asthmatic airway, thereby contributing to airway remodeling.

## List Of Abbreviations Used

αSMA: α-smooth muscle actin; BAL: bronchoalveolar lavage; DAPI: 4',6-diamidino-2-phenylindole; EMT: epithelial to mesenchymal transition; HRPO: horseradish peroxidase; IPF: idiopathic pulmonary fibrosis; LEF1: lymphoid enhancing factor 1; MMP: matrix-metalloproteinases; NHBE: normal human bronchial epithelial cells.

## Competing interests

The authors declare that they have no competing interests.

## Authors' contributions

AD performed all the experiments in the manuscript and participated in its design. BZ conceived of the study, and participated in its design and coordination and helped to draft the manuscript. All authors read and approved the final manuscript.
